# Better out than in: faecal matrix inhibits establishment success after waterfowl endozoochory

**DOI:** 10.1093/aob/mcaf192

**Published:** 2025-08-19

**Authors:** Iciar Jiménez-Martín, Andy J Green, Nándor Szabó, Balázs András Lukács, Orsolya Vincze, Ádám Lovas-Kiss

**Affiliations:** Department of Conservation Biology and Global Change, Estación Biológica de Doñana (EBD), CSIC, Sevilla 41092, Spain; Department of Conservation Biology and Global Change, Estación Biológica de Doñana (EBD), CSIC, Sevilla 41092, Spain; MTA-HUN-REN-CER ‘Momentum’ Dispersal Ecology Research Group, IAE, Debrecen 4026, Hungary; Wetland Ecology Research Group, HUN-REN Centre for Ecological Research, IAE, Debrecen 4026, Hungary; Wetland Ecology Research Group, HUN-REN Centre for Ecological Research, IAE, Debrecen 4026, Hungary; Littoral, Environnement et Sociétés (LIENSs), UMR 7266 CNRS, Université de La Rochelle, La Rochelle 17000, France; MTA-HUN-REN-CER ‘Momentum’ Dispersal Ecology Research Group, IAE, Debrecen 4026, Hungary; Department of Planetary Health, One Health Institute, Faculty of Health Sciencies, University of Debrecen, Debrecen 4032, Hungary

**Keywords:** Dispersal, *Eleocharis palustris*, germination, gut passage, *Juncus bufonius*, plant performance, seed dispersal, seedling establishment

## Abstract

**Background and Aims:**

Many plant species undergo long-distance dispersal through migratory waterbirds. However, there is little information about the effectiveness of this dispersal, especially regarding the chances of plant establishment and the impact of gut passage or the faecal matrix on plant germination, growth and reproductive investment in seeds.

**Methods:**

In a greenhouse experiment, we addressed these questions using an annual mudflat species, *Juncus bufonius* (Juncaceae), and a perennial emergent aquatic species, *Eleocharis palustris* (Cyperaceae), whose seeds are dispersed by many waterbird species in Europe. We planted seeds directly in soil or within mallard faeces placed on soil, using both control seeds and seeds that survived gut passage through mallards. Over the following 11 weeks, we quantified germination and plant performance.

**Key Results:**

Gut passage reduced germination time of *J. bufonius* when there was no faecal matrix, and it increased asymptotic height of *E. palustris*. Presence of the faecal matrix hindered germinability, plant growth and final biomass for both species, along with total seed production for *J. bufonius*. Presence of the faecal matrix slowed down germination in *E. palustris*, but had the opposite effect for *J. bufonius*. It was also associated with greater relative investment in seeds in *J. bufonius* (more seeds per unit biomass), probably as a consequence of later germination. In both species, earlier germination increased final biomass (and seed production in *J. bufonius*).

**Conclusions:**

Our results support the importance of waterbird endozoochory in plant dispersal but suggest that it might be more effective when faeces disintegrate, such as when egested into water or disaggregated on land (e.g. by insects). Previous studies with other plants have recorded accelerated germination following waterbird gut passage, and our results show that this can benefit plant fitness.

## INTRODUCTION

Long-distance dispersal of seeds allows plants to escape from competition and natural enemies and to colonize new areas (Beckman and Sullivan, 2023), with internal dispersal of seeds by animals (i.e. endozoochory) contributing significantly to this process. Waterfowl (Anatidae) and other waterbirds have been shown to be important dispersers of both aquatic and terrestrial plants (Green *et al.*, 2023), playing a key role in long-distance dispersal. During migration, they can provide extreme dispersal distances favoured by their long gut retention times (e.g. García-Álvarez *et al.*, 2015; Lovas-Kiss *et al.*, 2023*a*). Their daily movements of as much as 100 km or more (Martín-Vélez *et al.*, 2021*a*; Jiménez-Martín *et al.*, 2024) can connect different wetland habitats and thus provide directed long-distance dispersal for plants they disperse to favourable sites (Kleyheeg *et al.*, 2017). Habitat selection and movement patterns of the disperser are important for a successful dispersal event, which is also influenced by the patterns of faecal deposition and composition (Schupp, 1993; van Leeuwen *et al.*, 2022). As shown by Almeida *et al.* (2022, 2025), waterfowl with different feeding habits disperse different kinds of seeds, e.g. grazing geese disperse a higher proportion of seeds from terrestrial plants, whereas diving ducks disperse mainly hydrophyte plant species, favouring the matching of the source and destination habitats.

For an endozoochory seed dispersal event to succeed, the seed must first pass through the gut of the disperser and remain viable. Evidence is mounting that seeds from non-fleshy fruits, which account for the majority of those dispersed by waterbirds, can be adapted to survive gut passage (Costea *et al.*, 2019; Kreitschitz *et al.*, 2025). Although the benefits of gut passage for enhancement of germination are clear for fleshy-fruited species (Rogers *et al.*, 2021), contrasting results have been found from different studies on the effect of endozoochory by waterbirds on germination of non-fleshy-fruited plants (García-Álvarez *et al.*, 2015; van Leeuwen *et al.*, 2023; Lovas-Kiss *et al.*, 2023*b*).

There has been little research on the effect of gut passage by waterbirds on the development of adult plants after seed germination. Gut passage had no effect on growth and reproductive investment of *Potamogeton pectinatus* in mesocosms (Figuerola *et al.*, 2005), but reduced survival of *Ruppia maritima* in the field, owing to longer exposure to herbivory because of earlier germination (Figuerola and Green, 2004). These studies used intact clean seeds for sowing, which might simulate conditions when waterbirds egest into water. However, in natural conditions seeds are also often deposited imbedded in faeces, because birds often egest when walking or resting on, or flying over, land. There are no studies using waterbird droppings, but dung from different mammalian herbivores can affect development of dry-fruited plants. Negative effects on germination success (Cosyns *et al.*, 2005; Milotić and Hoffmann, 2016*b*) and positive effects on growth and flowering have been recorded for seeds sown in ungulate dung (Milotić and Hoffmann, 2016*a*). Seeds dispersed by bears also had lower germinability and longer germination time when embedded in the faecal matrix (Pauly *et al.*, 2025).

In the present study, we aimed to investigate the effects of endozoochorous seed dispersal by waterbirds on the different stages of plant development, using mallards (*Anas platyrhynchos*) as our model animal species. The mallard is a widespread waterfowl species and the most studied seed-dispersing waterbird in Europe, where it is known to disperse seeds of ≥240 different plant species (Almeida *et al.*, 2022). Its role as a seed disperser overlaps with that of many other waterbird species, especially other dabbling ducks (Green *et al.*, 2023; Almeida *et al.*, 2025). We used *Juncus bufonius* agg. and *Eleocharis palustris* as model plant species. Both species were selected because their seeds are dispersed by many waterbird species in Europe (Martín-Vélez *et al.*, 2021*b*; Almeida *et al.*, 2022). Our specific objectives were to investigate the effect of gut passage and the faecal matrix on seed germination, plant growth and reproductive investment in seeds. We hypothesized that: (1) gut passage would enhance germination, increasing the germinability and reducing time to germination; (2) the presence of waterbird faeces surrounding the seed would hinder germination, with seeds in faeces germinating less and later, but then the increased nutrient supply would later improve plant growth and reproductive investment; and (3) early germination would translate into greater plant mass and fecundity.

## MATERIALS AND METHODS

### Plant species

Two herbaceous cosmopolitan species native to Eurasia, North Africa and North America (Cope and Stace, 1978) and commonly dispersed by waterbirds were selected for the experiment. The toad rush, *Juncus bufonius* agg. (Juncaceae), is an annual species ≤40 cm high (Cope and Stace, 1978), whose fruits are capsules containing 0.3–0.5 mm seeds, which do not exhibit dormancy. Its Ellenberg *F*-value is seven, preferring moist soils (Julve, 1998). It can be found in all kinds of habitats with a high water table, occupying marshes and margins of rivers, streams, lakes and ponds. It can deal with brackish conditions.

The common spike-rush, *Eleocharis palustris* (Cyperaceae), is a perennial species ≤60 cm tall (Bojnanský and Fargašová, 2007), with a spikelet at the top of the stem containing 20–80 flowers, each of which produces an achene 1.2–2 mm long (Jiménez-Mejías and Luceño, 2008). According to Walters (1949), its seeds have a dormancy period of several months. Its Ellenberg *F*-value is 10 (Sonkoly *et al.*, 2023), occupying areas with shallow water, but it has rhizomes and is drought tolerant. The Ellenberg N indicators of soil fertility are five for *J. bufonius* and four for *E. palustris* plants (Hill *et al.*, 1999), indicating that they prefer soils of moderate fertility.

### Seed collection and storage

Given that duck species generally ingest seeds from the seed bank (Guillemain *et al.*, 2002; Urgyán *et al.*, 2023), we originally aimed to extract seeds from wetland seed banks. For this reason, *E. palustris* seeds were extracted from soil collected in Bihari-sík landscape protection area (Hungary) in January 2023 and were stored in the fridge wet until the experiment. However, drought conditions in Hungary at the time of our study made *J. bufonius* rarer than usual, and their seeds were not present in high enough densities in the wetlands of eastern Hungary to make it practical to extract seeds from seed banks. For this reason, we used *J. bufonius* seeds collected in April 2022 in Doñana ricefields (Spain), directly from five individual plants of the same population, that were stored dry at a constant temperature of 22 °C until the experiment. Seeds of a given species were thoroughly mixed before random assignment to different experimental treatments.

### Feeding trial

The mallard is widely reared in captivity. Eight mallards (six females and two males), aged 1 year, were purchased from a local breeder for the feeding experiment. The mallards were confined in individual wire mesh cages (60 cm × 40 cm × 50 cm). They were kept together in the same room and had visual contact with each other. Plastic trays were placed under each cage to collect faeces during the experiment. Water and food (grains of corn, oat and wheat mixture) were offered *ad libitum* during confinement. Their faeces were also collected during 3 days before the feeding trial started, and these faecal samples were stored wet in the fridge (4 °C), and then used for the different sowing treatments (pre-trial faeces, see below).

On 18 April 2023, each mallard was force-fed with 100 seeds of *E. palustris* and 400 seeds of *J. bufonius* mixed together in a small bread ball. After ingestion of seeds, faecal samples were collected at specific time intervals: every 2 h during the first 8 h, then 12 and 24 h after feeding, and they were stored in the fridge until processing. Faeces were processed to extract the seeds as soon as possible, over the 5 days following the feeding trial. Faeces were washed through a 100 μm sieve and inspected under a stereomicroscope (Alpha STO-4TLED) in Petri dishes. Intact retrieved seeds found (Supplementary Data Table S1) were stored in the fridge (4 °C) until sowing.

### Sowing and treatments

Seeds were sown between 3 and 6 May 2023 in four different treatment groups: control (non-ingested seeds) with addition of faeces (CF), control seeds with no faeces (CNF), ingested seeds with addition of faeces (IF) and ingested seeds without faeces (INF). Among the seeds retrieved from mallard droppings, we sowed 233 (IF) and 232 (INF) *J. bufonius* seeds and 159 (IF) and 160 (INF) *E. palustris* seeds. Among control seeds, we sowed 50 seeds in CNF, whereas in the CF treatment we divided them into eight sets, with six seeds per set (i.e. we sowed 48 in total) and applied pre-trial faeces from a different individual mallard to each of these sets.

Perforated plastic pots (diameter, 5.5 cm; height, 6 cm) were half-filled with soil collected from the Bihari-sík landscape protection area, which was completely dried and sterilized before the experiment, then watered before sowing. For treatments including addition of faeces, a piece of pre-trial faeces (<1 cm thick) was placed on top of the soil in the centre of each pot, and the seeds were inserted with tweezers one by one into the faeces at a shallow depth. In CNF and INF treatments, seeds were inserted directly into the soil surface at a similar shallow depth. In the IF treatment, each retrieved seed was sown with pre-trial faeces from the same individual mallard that ingested it. Only one seed was sown per pot, and all pots were placed randomly in plastic trays in an outdoor greenhouse with natural light. During the experiment, the daily minimum and maximum temperatures inside the greenhouse were 16.8 ± 3 and 41.2 ± 9.4 °C (mean ± s.d.), respectively. The daily minimum and maximum relative humidity levels were 27.5 ± 15.9 and 78.5 ± 10.9 % (mean ± s.d.), respectively.

### Monitoring

Germination was monitored during the first month, checking the pots for new seedlings daily, and seeds and plants were watered daily by filling the trays with water in order to avoid physical disturbance. Plant height was measured on the 7th and 15th days after germination, then every 15 days until the 45th day. We also took measurements on the 60th day, but an important fraction of plants died beforehand, hence we did not use these data in our analyses. Height was measured with a tape measure, the end of which was placed on top of the soil, and the height of the longest leaf was measured. Plants were collected 70–78 days after sowing, when we observed the signs of senescence in several plants, probably related to the high summer temperatures reached in the greenhouse at that time (>45 °C). For the plants of *J. bufonius* (annual species), the number of fruits was counted at the time of collection. When possible, given that many fruits had already opened and spread their seeds, we collected three closed fruits per plant and counted the number of seeds per fruit. These data were used to estimate the total number of seeds per plant by multiplying the average number of seeds per fruit by the total number of fruits on the plant. In contrast to *J. bufonius*, no fruiting variables were determined for *E. palustris* because this perennial did not flower during the experiment. After collection, plants were dried for 48 h, and the above-ground part was separated from the roots and weighed in a balance with a precision of 0.0001 g.

### Statistical analyses

All data management and statistical analyses were performed in R software, v.4.2.1 (R Core Team, 2024). The two plant species were analysed separately. Data on seed retention time (i.e. time elapsed between force-feeding and egestion) was not included in the analyses because the number of seeds recovered per time interval were uneven, with most seeds egested within the first 6 h (Supplementary Data Table S1). Nonetheless, we made sure to allocate seeds passed at different times in equal proportions among the experimental groups.

For germination variables (germinability and time to germination), generalized linear models (GLMs) were fitted to test the effect of faeces and gut passage treatments on each response variable. Germination was analysed as a binary response (yes or no) variable in models with a binomial error distribution, including faeces treatment and gut passage treatment as fixed explanatory effects. Time to germination was used as a dependent variable in models with Poisson or negative binomial error distributions, and the same fixed effects as for germination. A negative binomial error distribution was applied only if overdispersion was detected in the Poisson model.

In order to assess differences in growth patterns among treatments, we modelled plant height across time by fitting non-linear mixed effects models with a three-parameter logistic base function, following a procedure similar to Milotić and Hoffmann (2016*a*). The three-parameter logistic function is commonly applied to model plant growth data (Paine *et al.*, 2012):


$$H_{t}=\frac{\emptyset_{1}}{1+\exp\;\left(\frac{-(t-\emptyset_{2})}{\emptyset_{3}}\right)}$$

In this function, *H* is the plant height at time *t*, while Ø_1_ (asym), Ø_2_ (xmid) and Ø_3_ (scal) are parameters defining the growth curve (Pinheiro and Bates, 2000). The asymptotic height (Ø_1_) indicates the estimated maximum height reached by the plant. Xmid (Ø_2_) is the time at which the plant reaches 0.5Ø_1_ and refers to how quickly the plant grows at the beginning and reaches an important size, with lower values indicating a faster growth. Scal (Ø_3_) is the time elapsed between the plant reaching half and three-quarters of Ø_1_ and is a measure of the steepness of the curve, with lower values indicating a steeper curve and therefore a faster growth rate during this intermediate phase. The time unit was days after germination. We used the *nlme* package (Pinheiro *et al.*, 2024) to fit separate models for each plant species, with height as the response variable. Given that multiple measurements of the same individual plants were entered in these models, we included plant identity as a random factor to control for the non-independence of the measurements. Faeces treatment and gut passage treatment were included as fixed effects in these models to test their influence on the three estimated model parameters, thus evaluating how they affect the pattern of growth in plant height.

We then assessed the effect of faeces and gut passage treatments on plant above-ground mass at collection time, while controlling for time to germination. To explore this question, we built linear regression models with faeces treatment, gut passage treatment and time to germination as fixed effects, and with above-ground mass as the response variable. For *E. palustris*, the residuals from the simple linear regression model showed a curved pattern and deviated from normality, suggesting a quadratic relationship between above-ground mass and time to germination. Thus, to capture this curved relationship, we included a quadratic term for germination time in the model, which satisfied model assumptions of normality, linearity and homoscedasticity.

To study whether adding faeces served as a source of nutrients to increase seed production while controlling for plant mass, the relationship between the number of seeds per plant and above-ground mass at collection time was modelled using a standardized major axis (SMA) regression with the *smatr* package (Warton *et al.*, 2012). This technique is appropriate when the objective is not to predict or test for an association, but to estimate the line that best describes the relationship between two variables, especially when there is error in the *x-variable* (Warton *et al.*, 2006). We added the faeces addition × plant above-ground mass interaction to fit two different regression lines, one for plants with faeces addition and another for those without. Then, we tested for common slopes between the two lines to check whether the relationship between the number of seeds and plant mass differed among plants with and without addition of faeces. If the slopes did not differ, we tested for equal elevations (i.e. intercepts) between the two lines (Warton *et al.*, 2006). The normality and distribution of residuals were visually inspected for models with and without log-transformation, and the one presenting a distribution closer to normality was chosen. Then, to assess the differences in the total number of seeds per plant among treatments (without controlling for plant mass), we fitted a generalized linear model with a negative binomial error distribution with the number of seeds as the response variable and with gut passage treatment, faeces treatment and time to germination as the explanatory variables.

All multivariate models involving gut passage and faeces variables were fitted with and without their first-order interaction. Then, these different model pairs were compared using a likelihood ratio test to assess whether the more complex model provided a significantly better fit than the simpler model (Supplementary Data Table S2). The comparison was performed with the *anova* function, and if the models were not significantly different we present the simpler model (without the interaction). Model residuals were examined for potential heteroscedasticity, overdispersion and zero-inflation with the package *DHARMa* (Hartig, 2022). We assessed model fits by calculating the *r*^2^ for linear models or the Nagelkerke pseudo-*r*^2^ (Nagelkerke, 1991) for GLMs with the *nagelkerke* function from the rcompanion package (Mangiafico, 2024). After fitting the models, if the interaction faeces × gut passage was significant, we performed *post hoc* pairwise comparisons with Tukey’s honest significant difference test using the *emmeans* function from the *emmeans* package (Lenth, 2024) to test for statistical differences among the different treatments.

Finally, in order to test whether the effect of the two treatments could vary among different mallard individuals, generalized linear mixed models were fitted with only ingested seeds, including faeces treatment as a fixed effect and mallard ID (factor, eight mallards, controlling for which individual egested the seed and also provided the faeces in which it was sown) as the random term. We fitted these models with different dependent variables: germination; time to germination; above-ground mass; and seed production. Each model was compared with an equivalent GLM without the random term using likelihood ratio tests to test whether adding the random term mallard identity significantly improved the model fit. Additionally, we checked whether the effect of faeces had the same sign and level of significance within each pair of models, which were consistent in every case (Supplementary Data Table S3).

## RESULTS

We retrieved 324 *E. palustris* seeds and 1486 *J. bufonius* seeds from the droppings (Supplementary Data Table S1), representing 41 and 46 % of the seeds fed to the mallards, respectively. A total of 417 *E. palustris* and 563 *J. bufonius* seeds were sown, of which 224 (54 %) and 323 (57 %) germinated. Some plants died before day 30 after germination: for *E. palustris*, two in INF; and for *J. bufonius*, two in INF, three in CF and eight in IF treatments. These plants were included only in analyses of germination.

### Effects of gut passage and faeces addition on germination patterns

**Fig. 1.**
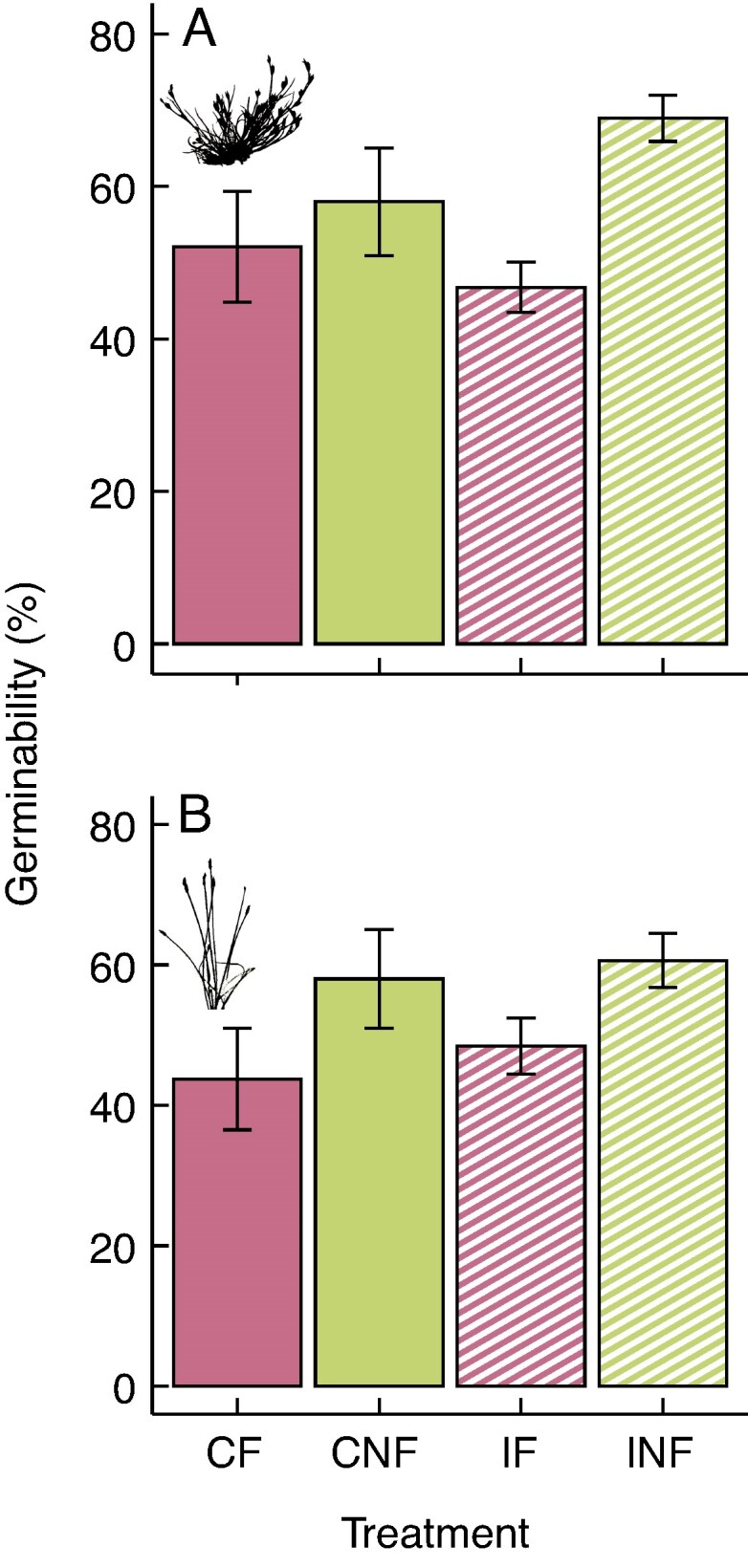
Germinability of *Juncus bufonius* (A) and *Eleocharis palustris* (B) seeds per treatment. Bars represent means ± s.e. Treatments including faeces addition are in pink (CF and IF), and treatments including seed ingestion are represented with dashed lines (IF and INF). Abbreviations: CF, control, faeces; CNF, control, no faeces; IF, ingested, faeces; INF, ingested, no faeces. Faeces had a significant effect in both species, but gut passage did not (Supplementary Data Table S4).

**Fig. 2.**
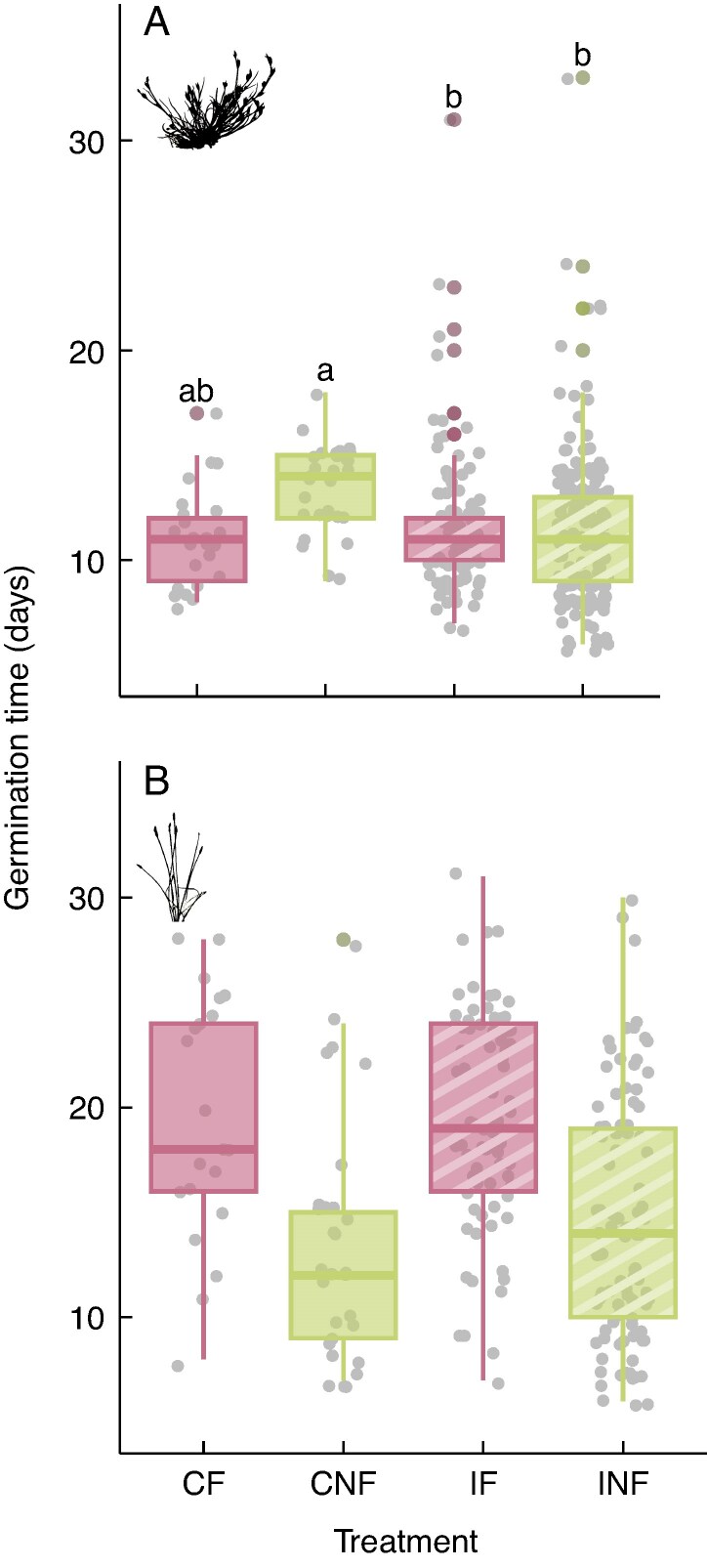
Time to germination per treatment for *Juncus bufonius* (A) and *Eleocharis palustris* (B) seeds. Treatments including faeces addition are in pink (CF and IF), and treatments including gut passage are represented with dashed lines (IF and INF). The line within each box represents the median, the lower and upper edges correspond to the first and third quartiles, the upper and lower whiskers extend to the largest and smallest value no further than ±1.5 × interquartile range, and individual coloured data points outside the whiskers are outliers. Treatments that do not share any letter differ significantly (*P* < 0.05) based on *post hoc* tests from the *J. bufonius* generalized linear model of Supplementary Data Table S5. For *E. palustris*, faeces had a significant effect (Supplementary Data Table S5). Abbreviations: CF, control, faeces; CNF, control, no faeces; IF, ingested, faeces; INF, ingested, no faeces.

Addition of faeces significantly reduced the germinability of the seeds from both species, whereas gut passage increased it, albeit not significantly (Fig. 1; Supplementary Data Table S4). In the germination time model for *J. bufonius* seeds, there was a significant interaction between faeces and gut passage (*P* = 0.023; Supplementary Data Table S5A), with control seeds without faeces (CNF) taking longer to germinate than ingested seeds or CF seeds (Fig. 2A). *Eleocharis palustris* seeds germinated significantly later when sown with addition of faeces (*P* < 0.001; Fig. 2B; Supplementary Data Table S5B), and gut passage slightly delayed germination in a non-significant way. This model was the only one showing an effect of mallard identity (Supplementary Data Table S3), with INF seeds from one of the mallards showing delayed germination in comparison to others (18.9 ± 5.52 vs. 21 ± 3.46 days; mean ± s.d.).

### Effects of gut passage, addition of faeces and time to germination on plant growth

*Juncus bufonius* plants were growing for a mean (±s.d.) of 61 ± 4 days (range = 54–72 days) before harvesting, and *E. palustris* plants for a mean (±s.d.) of 59 ± 5 days (range = 47–73 days).

**Fig. 3.**
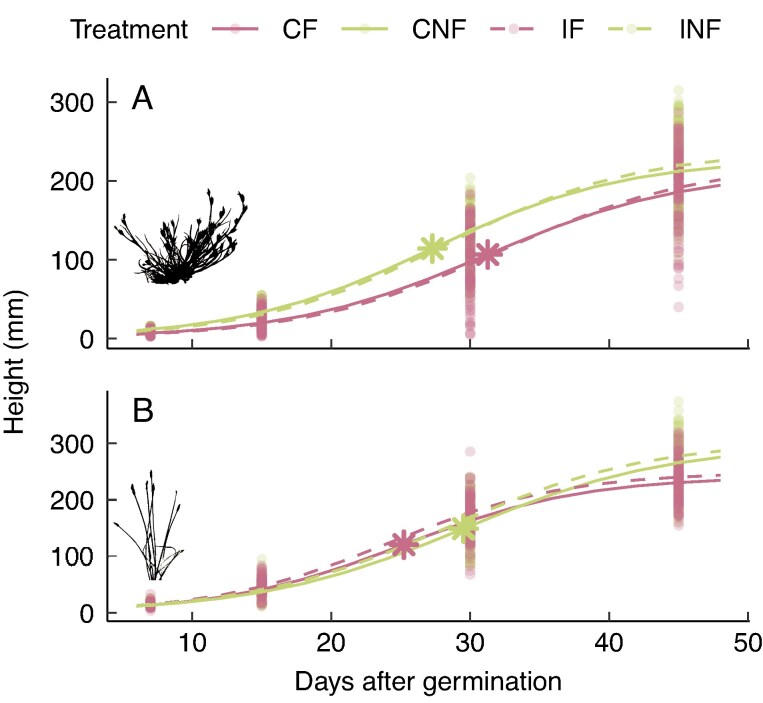
Plant height measured at 7, 15, 30 and 45 days after germination for *Juncus bufonius* (A) and *Eleocharis palustris* (B). Curves were obtained from the fitted logistic base functions (Table 1), and points represent measured height for individual plants. Treatments including faeces addition (CF and IF) are represented in pink, and treatments including gut passage (IF and INF) with dashed lines. Asterisks represent mean xmid parameters for treatments grouped with faeces (pink) and without faeces (green). Abbreviations: CF, control, faeces; CNF, control, no faeces; IF, ingested, faeces; INF, ingested, no faeces.

Addition of faeces significantly decreased the asymptotic height reached by *J. bufonius* (Asym; Table 1A; Fig. 3A) and decreased the time needed to fulfil the second part of the growth curve for *E. palustris* (scal; Table 1B; Fig. 3B), by increasing the growth rate during this intermediate phase. Ingestion significantly increased the final height reached by *E. palustris* (Asym; Table 1B), with plants in the two treatments including gut passage (IF and INF) achieving a greater height than the respective control treatments (CF and CNF; Fig. 3B).

**Table 1.** Results of nonlinear mixed effects growth models using three parameter logistic base curves with plant height as the response variable, plant id as the random term and gut passage and faeces addition as fixed effects for (A) *Juncus bufonius* (*N* = 305 plants) and (B) *Eleocharis palustris* (*N* = 212 plants).

	*Species*	*Parameter*	*Predictor*	*Estimate*	*s.e.*	*F-value*	*P*-value
*(A)*	*Juncus bufonius*	Asym (Ø_1_)	Intercept	228.4	7.441	–	–
			Faeces (addition)	−15.219	6.105	42.944	**<0.001**
			Gut passage (ingested)	10.120	7.748	0.517	0.472
		Xmid (Ø_2_)	Intercept	27.282	0.810	–	–
			Faeces (addition)	3.978	0.641	1.001	0.317
			Gut passage (ingested)	0.967	0.837	0.002	0.961
		Scal (Ø_3_)	Intercept	6.936	0.182	–	–
			Faeces (addition)	0.200	0.137	2.193	0.139
			Gut passage (ingested)	−0.073	0.186	0.155	0.694
*(B)*	*Eleocharis palustris*	Asym (Ø_1_)	Intercept	298.152	11.447	–	–
			Faeces (addition)	−59.709	7.634	0.147	0.702
			Gut passage (ingested)	7.634	11.732	10.252	**0.001**
		Xmid (Ø_2_)	Intercept	29.523	1.029	–	–
			Faeces (addition)	−4.293	0.874	0.389	0.533
			Gut passage (ingested)	−0.988	1.072	0.304	0.582
		Scal (Ø_3_)	Intercept	7.393	0.238	–	**–**
			Faeces (addition)	−0.926	0.203	20.912	**<0.001**
			Gut passage (ingested)	−0.190	0.248	0.583	0.445

Final models were selected based on likelihood ratio tests comparing models with and without a gut × faeces interaction (Supplementary Data Table S2). Significant *P*-values are marked in bold. Asym (Ø_1_) is the asymptotic height, xmid (Ø_2_) is the time at which the plant reaches 0.5Ø_1_, and scal (Ø_3_) is the time elapsed between the plant reaching half and three-quarters of Ø_1_. Predictor levels without ingestion or faeces addition are aliased in the model (i.e. estimates are effectively zero). For the fitted curves, see Fig. 3.

**Fig. 4.**
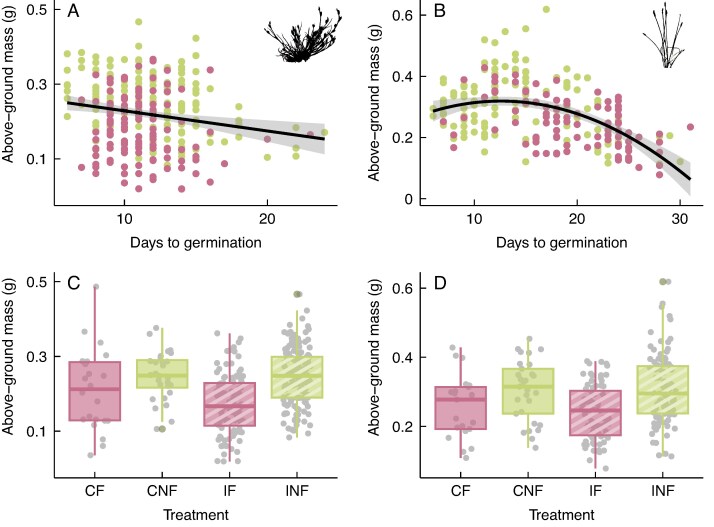
Effects of time to germination (A, B) and treatment (C, D) on above-ground mass for *Juncus bufonius* (A, C) and *Eleocharis palustris* (B, D) plants. Treatments including faeces addition (CF and IF) are represented in pink, and treatments including ingestion (IF and INF) with dashed lines. Fitted lines and their standardized errors represent the linear (A) and quadratic (B) models fitted to the entire dataset, with pink and green dots indicating data points for plants with and without faeces addition, respectively. The line within each box (C, D) represents the median, the lower and upper edges correspond to the first and third quartiles, the upper and lower whiskers extend to the largest and smallest value no further than ±1.5 × interquartile range, and individual coloured data points outside the whiskers are outliers. Abbreviations: CF, control, faeces; CNF, control, no faeces; IF, ingested, faeces; INF, ingested, no faeces.

For both species, plants germinating later reached a significantly lower final above-ground mass (Fig. 4A, B; Supplementary Data Table S6). In *E. palustris* plants, this effect was most notable for plants germinating after 11 days, which corresponds to the germination time at which above-ground mass peaked. For plants germinating earlier, days to germination showed a slightly positive relationship with above-ground mass (Fig. 4B). Addition of faeces also had a significant negative partial effect on final plant above-ground mass, with IF plants reaching the lowest median mass (Fig. 4C, D). A negative partial effect of gut passage on final mass was not statistically significant (Supplementary Data Table S6).

### Effects of gut passage and addition of faeces on J. bufonius seed production

**Fig. 5.**
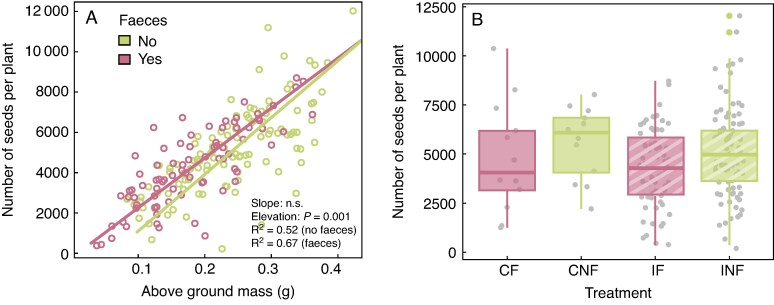
Effects of faeces and gut passage on *Juncus bufonius* seed production. (A) Standardized major axis regression between above-ground mass and the number of seeds per plant for plants sown with (*y* = −221.35 + 24 769.46*x*) and without (*y* = −1623.69 + 27 999.30*x*) addition of faeces (*n* = 189 plants). Significance test results are shown for standardized major axis (SMA) slopes and SMA elevations (i.e. intercepts), in addition to *R*^2^ values. (B) Number of seeds produced per plant per treatment. The line within each box represents the median, the lower and upper edges correspond to the first and third quartiles, the upper and lower whiskers extend to the largest and smallest value no further than ±1.5 × interquartile range, and individual coloured data points outside the whiskers are outliers. Abbreviations: CF, control, faeces; CNF, control, no faeces; IF, ingested, faeces; INF, ingested, no faeces.

We were able to estimate the total number of seeds produced for 189 *J. bufonius* plants. The SMA regression showed that the slope between the number of seeds per plant and above-ground mass did not differ among treatments with and without added faeces (Fig. 5A). However, the elevation (*y*-intercept) was significantly different, with more seeds per plant for a given above-ground mass in plants sown with faeces (Fig. 5A). This difference was not pronounced for larger plants, >0.25 g (Fig. 5A). However, the results from a negative binomial model without controlling for plant mass showed that addition of faeces and late germination both had a significant negative partial effect on the number of seeds, whereas the partial effect of gut passage was not significant (Fig. 5B; Supplementary Data Table S7). Hence, the faecal matrix reduced total seed production by delaying germination, but increased the number of seeds per gram of above-ground mass.

## DISCUSSION

We evaluated the effects of waterbird endozoochory, from seed ingestion to seed deposition, on the early stages of plant establishment of a perennial plant (*E. palustris*) and on the whole cycle until seed production of an annual plant (*J. bufonius*). Overall, gut passage of seeds by mallards did not show major effects on plant germination and development, whereas the addition of faeces showed various negative effects. Except for germination time in *J. bufonius*, we found no evidence of interactions between gut passage and the presence of faeces.

### Influence of gut passage and the faecal matrix on germination

Some previous studies reported higher germinability after seed ingestion by waterbirds (Figuerola *et al.*, 2005; Brochet *et al.*, 2010; Nie *et al.*, 2022) and others reported mainly a negative effect (Wongsriphuek *et al.*, 2008; García-Álvarez *et al.*, 2015; van Leeuwen *et al.*, 2023), whereas germinability was not significantly affected by gut passage in the present study. Differences among studies might be explained, in part, by differing ability of gut passage to break seed dormancy (Espinar *et al.*, 2023). Our results might have been sensitive to the duration of the interval after seed collection, the way in which seeds were stored before the experiment, and the difference between extracting seeds from the seed bank (for *E. palustris*) or taking them from mother plants (for *J. bufonius*). *Eleocharis palustris* seeds were collected 4 months before the experiment, in January. The cold winter temperatures to which the seeds were exposed, followed by storage in cold conditions after collection, might already have broken seed dormancy in these seeds, making the effect of ingestion insignificant. Brochet *et al.* (2010) stored *E. palustris* seeds after initially drying them, which might have induced dormancy, perhaps explaining why they found gut passage by teal *Anas crecca* to increase germinability and reduce germination time, whereas we found no effects for wet stored seeds. Seeds from *J. bufonius* would probably not need to break dormancy, because they have been reported to germinate during the whole vegetative season and in different environmental conditions (Carta *et al.*, 2013).

Addition of faeces decreased germinability by 24 % for *E. palustris* seeds and by 10 % for *J. bufonius* seeds. It has previously been seen that the presence of pellet material from barn owls (*Tyto alba*) increases germinability of *Sinapis alba* seeds (Godó *et al.*, 2023). Furthermore, several experiments have added herbivore dung to investigate dispersal of dry-fruited plants by mammals. Milotić and Hoffmann (2016*b*) tested the effects of cattle and horse dung on germination using 15 grassland species, including *J. bufonius*, and they found negative effects of both types of dung on the germinability of all species. Likewise, horse and Patagonian mara (*Dolichotis patagonum*) dung inhibited germination of *Neltuma flexuosa* seeds, but there were no effects of cow manure (Ramos *et al.*, 2024). More species and seedlings of dry-fruited plants germinated from brown bear scat samples from the wild when faeces were filtered, in comparison to intact faeces (Pauly *et al.*, 2025). Our study is generally consistent with mammalian studies, suggesting that the faecal matrix has a mainly negative effect on the germination of dry-fruited plants. However, in all these studies, including ours, the treatments with faeces addition were applied in controlled conditions without the biotic (e.g. insects) or abiotic disaggregation agents present in nature, which might facilitate germination of seeds imbedded in the faecal matrix (Pauly *et al.*, 2025). Furthermore, we sterilized the soil but not the faeces, meaning that fungi or bacteria, together with the toxic compounds present in the faeces, might have been detrimental for germination (Milotić and Hoffmann, 2016*b*). Although it has been shown that waterfowl faeces can boost soil fertility and growth of sub-arctic vegetation, fresh faeces volatize important amounts of ammonia (Ruess *et al.*, 1989). Ammonia is toxic and can inhibit seed germination (Bremner and Krogmeier, 1989; Wan *et al.*, 2016), potentially explaining our results.

The effects of gut passage and faeces treatments on time to germination differed considerably between the two plant species. Ingestion and addition of faeces accelerated germination of *J. bufonius* seeds, with plants in the CNF treatment germinating an average of 2 days later than seeds in any of the other treatments. Conversely, addition of faeces delayed germination of *E. palustris* seeds by 5 days. Previous studies show no consistent pattern in the effect of gut passage on germination time, with ingested seeds germinating later than controls in some plant species and earlier in others (van Leeuwen *et al.*, 2023). In our case, plants germinating later were generally at a disadvantage, reaching a lower final above-ground mass. However, whether earlier or later germination has any positive effect for plant establishment in nature depends on interactions with other species. If a plant germinates faster, it can have more time to grow and gain an advantage in plant competition, but it can also be exposed earlier to herbivores (Figuerola and Green, 2004) and pathogens (Traveset, 1998). Early germination might be more important for annual plants, such as *J. bufonius*, with rapid senescence in summer, because it allows them to extend their growing period. For a perennial plant, such as *E. palustris*, the carry-over effects of early germination for fruit production in the long term remain unclear.

### Influence of gut passage and addition of faeces on plant growth and reproduction

Growth patterns did not differ markedly among treatments, but the intermediate phase of growth was significantly faster in *E. palustris* plants with addition of faeces, although it was not reflected in the final height. In contrast, *E. palustris* plants from ingested seeds showed a higher asymptotic height, whereas Figuerola *et al.* (2005) found no such effect on a pondweed. In *J. bufonius*, addition of faeces significantly decreased the asymptotic height. Furthermore, both species reached a lower above-ground mass when sown with faeces, being reduced by 27 % in *J. bufonius* plants and 20 % in *E. palustris* plants, although faeces had accelerated germination of *J. bufonius* seeds. This is contrary to our initial hypothesis of an enhancement of growth of plants with addition of faeces, attributable to the nutrients provided. The effect of faeces might be context dependent and determined by soil quality (Ruess *et al.*, 1989), biotic agents and weather conditions (Pauly *et al.*, 2025). In our experimental conditions, with the absence of disaggregation agents, such as coprophages or rain, faeces played a detrimental role in the establishment of plants, because achieving greater height and mass has clear benefits for plant fitness, such as attracting more pollinators (Zu and Schiestl, 2017), being more exposed for wind pollination, achieving greater seed dispersal distances (Thomson *et al.*, 2011) or having better access to sunlight.

Plant fitness also depends on the reproductive output, which we estimated from the number of seeds produced by *J. bufonius*. The total number of seeds per plant was lower in plants with faeces, although the number of seeds per gram of above-ground mass was higher. Milotić and Hoffmann (2016*a*) found for the same plant species that the number of flowers and the number of flowers per gram of plant biomass were higher for control plants. The greater investment in seed production per unit biomass that we found with faecal addition might be related to stress. However, it seems most likely to be connected with the delayed germination of plants with faecal treatment, which put more investment into flowering to compensate for the reduced time for growth. Plants can prioritize the production of seeds over growth to ensure reproductive success. *Juncus bufonius* is a mudflat species, and increasing temperatures and reduced soil moisture in spring limit the growth period and prevent an extended flowering period. *Eleocharis palustris* is also common in temporary wetlands where the growth period is limited, although it also often occurs at the edge of wetlands with more stable water levels, where delayed germination might have lower costs.

Given the negative effects of the faecal matrix, establishment of dispersed seeds might be more likely when waterbirds egest their faeces when swimming or flying over water, because their faeces then rapidly disintegrate, quickly liberating intact seeds from within the matrix. Gut passage greatly reduces seed floatability (Navarro-Ramos *et al.*, 2024), hence these seeds are likely to sink quickly into the seed bank, where they might remain viable for many months or years until conditions become suitable for germination (Espinar *et al.*, 2023). Furthermore, the effects of the faecal matrix might be different for other plant species, such as those with higher nutrient requirements. The content of the faecal matrix is itself is likely to be important, and we fed mallards on a plant diet. Previous comparisons of animal and plant diet in captive mallards show that they can have quantitative effects on germination of seeds sown without faeces (Charalambidou *et al.*, 2005; Kleyheeg *et al.*, 2018). In the field, the diet of mallards and other omnivorous waterfowl, including the proportion of animal and plant material, varies considerably in space and time (Dessborn *et al.*, 2011), and further work is needed to address how changes in faecal composition can influence the effects on dispersed seeds. In addition, faeces from different waterbirds, such as herbivorous coots, geese or swans, or largely carnivorous gulls and storks (Martín-Vélez *et al.*, 2021*b*), might have a different composition from that of mallards and have different impacts on seed germination and plant growth. Nevertheless, any ‘endozoochory costs’ from deposition within a faecal matrix can be counterbalanced by the benefits of the long dispersal distances, and dispersal directed to suitable habitats, provided by birds (Kleyheeg *et al.*, 2017; Green *et al.*, 2023).

### Conclusion

The present study sheds light on dispersal steps and effectiveness following seed deposition after ingestion by waterbirds. In comparison to frugivorous birds, there has been little experimental investigation of seed dispersal by non-frugivorous birds. For two dry-fruited plants whose seeds are regularly dispersed by waterbirds, passage through the avian gut did not alter the germination and growth of seeds extracted from faeces. However, the faecal matrix had mainly harmful effects on plant development, and further research is needed to compare our results with those for different captive diets, in addition to those in more natural conditions. For example, the presence of abiotic and biotic disaggregation agents, more rapid volatilization of ammonia, or deposition in water might remove the inhibitory effects of the faecal matrix. Experiments with other waterbirds with different feeding habits, and with other plant species, are also required to establish general patterns of dispersal effectiveness for waterbird endozoochory.

## Supplementary Material

mcaf192_Supplementary_Data

## SUPPLEMENTARY INFORMATION

Supplementary data are available at *Annals of Botany* online and consist of the following. Table S1: intact seeds retrieved from mallard faeces per time interval. Table S2: results of likelihood ratio tests comparing models with and without a gut × faeces interaction. Table S3: results of likelihood ratio tests comparing models with and without mallard identity as a random term, including only seeds from ingestion treatments. Table S4: results from *Juncus bufonius* and *Eleocharis palustris* generalized linear models fitted with a binomial error distribution testing the effects of gut passage and faecal treatments on germinability. Table S5: results from *Juncus bufonius* and *Eleocharis palustris* generalized linear models testing the effects of gut passage and faecal treatments on germination time. Table S6: results from *Juncus bufonius* and *Eleocharis palustris* simple linear regressions examining the effects of time to germination, faeces and gut passage on above-ground plant biomass at collection time. Table S7: results from the negative binomial model testing the effect of gut passage, faeces addition and time to germination on the total number of seeds per plant for *Juncus bufonius*.
