# Supplementary material for: Better out than in: faecal matrix inhibits establishment success after waterfowl endozoochory
Source: Ann Bot. 2025 Aug 19;136(7):1601–11. doi: 10.1093/aob/mcaf192 (PMC12718015; doi:10.1093/aob/mcaf192)
Supplement: mcaf192_Supplementary_Data [file mcaf192_supplementary_data.docx]

**Supplemental Information for:**

**Better out than in: faecal matrix inhibits establishment success after waterfowl endozoochory**

**Authors:** Iciar Jiménez-Martín, Andy J. Green, Nándor Szabó, Balázs András Lukács, Orsolya Vincze, Ádám Lovas-Kiss

**Table S1.** Intact seeds retrieved from mallard faeces per time interval.

**Table S2.** Results of likelihood ratio tests comparing models with and without a gut x faeces interaction.

**Table S3.** Results of likelihood ratio tests comparing models with and without mallard ID as a random term, including only seeds from ingestion treatments.

**Table S4.** Results from *J. bufonius* and *E. palustris* generalized linear models fitted with a binomial error distribution testing the effects of gut passage and faecal treatments on germinability.

**Table S5.** Results from *J. bufonius* and *E. palustris* generalized linear models testing the effects of gut passage and faecal treatments on germination time.

**Table S6.** Results from *J. bufonius* and *E. palustris* simple linear regressions examining the effects of time to germination, faeces and gut passage on above-ground plant biomass at collection time.

**Table S7**. Results from the negative binomial model testing the effect of gut passage, faeces addition and time to germination on the total number of seeds per plant for *J. bufonius.*

**Table S1.** Intact seeds retrieved from mallard faeces per time interval. These are the total numbers of seeds retrieved, and not all of them were sown.

| Interval | *J. bufonius* | *E. palustris* |
| --- | --- | --- |
| 0 – 2 h | 416 | 83 |
| 2 – 4 h | 1048 | 193 |
| 4 – 6 h | 13 | 8 |
| 6 – 8 h | 7 | 13 |
| 8 – 12 h | 2 | 5 |
| 12 – 24 h | 0 | 22 |

**Table S2.** Results of likelihood ratio tests comparing models with and without a gut x faeces interaction.

| Species | Model | *χ^2^* | p |
| --- | --- | --- | --- |
| *E. palustris* | Germination | 0.029 | 0.865 |
| *J. bufonius* | Germination | 2.318 | 0.128 |
| *E. palustris* | Germination time | 0.491 | 0.483 |
| *J. bufonius* | Germination time | 5.254 | **0.022** |
| *E. palustris* | Growth | 2.426 | 0.489 |
| *J. bufonius* | Growth | 7.019 | 0.071 |
| *E. palustris* | Above ground mass | 0.769 | 0.382 |
| *J. bufonius* | Above ground mass | 0.691 | 0.407 |
| *J. bufonius* | Seed production | 0.068 | 0.794 |

**Table S3**. Results of likelihood ratio tests comparing models with and without mallard ID as a random term, including only seeds from ingestion treatments. Faeces treatment was included as the fixed effect and if its effect was significant in both models and with a negative sign, an asterisk is marked after the name in the model column. If faeces effect was significant and with a positive sign in both models, it is marked with ^+^. If faeces treatment was not statistically significant in any of the models, no symbol is added. The effect of the faeces treatment did not differ from the one reported in the main text (i.e. including non-ingested seeds and the other fixed effects) for any of the models. CI: confidence interval for the random term (variance).

| Species | Model | Family | *χ^2^* | p | 2.5 – 97.5% CI |
| --- | --- | --- | --- | --- | --- |
| *E. palustris* | Germination* | Binomial | 0.582 | 0.446 | 0 – 0.659 |
| *J. bufonius* | Germination* | Binomial | 1.744 | 0.187 | 0 – 0.633 |
| *E. palustris* | Germination time^+^ | Neg. binomial | 14.427 | **0.001** | 0.053 – 0.226 |
| *J. bufonius* | Germination time | Poisson | 0 | 1 | 0 – 0.072 |
| *E. palustris* | Above ground mass* | Linear regression | 1.146 | 0.284 | 0 – 0.042 |
| *J. bufonius* | Above ground mass* | Linear regression | 3.253 | 0.071 | 0 – 0.031 |
| *J. bufonius* | Seed production | Neg. binomial | 0 | 1 | 0 – 0.135 |

**Table S4**. Results from (a) *J. bufonius* (N = 563 seeds) and (b) *E. palustris* (N = 417) generalized linear models fitted with a binomial error distribution testing the effects of gut passage and faecal treatments on germinability*.* Final models were selected based on likelihood ratio tests comparing models with and without a gut x faeces interaction (Table S2). Significant p-values are highlighted in bold. Predictor levels without ingestion or faeces addition are aliased in the model (i.e. estimates are effectively zero).

|  | **Species** | **Response variable** | **Predictor** | ***Estimate*** | ***SE*** | ***χ^2^*** | ***p*** | ***R^2^*** |
| --- | --- | --- | --- | --- | --- | --- | --- | --- |
| (a) | *Juncus bufonius* | Germinability | Intercept | 0.606 | 0.225 |  |  | 0.051 |
|  |  |  | Gut passage (ingested) | 0.126 | 0.228 | 0.302 | 0.583 |  |
|  |  |  | Faeces (addition) | -0.803 | 0.174 | 21.717 | **<0.001** |  |
| (b) | *Eleocharis palustris* | Germinability | Intercept | 0.293 | 0.226 |  |  | 0.023 |
|  |  |  | Gut passage (ingested) | 0.148 | 0.233 | 0.404 | 0.525 |  |
|  |  |  | Faeces (addition) | -0.513 | 0.198 | 6.765 | **0.009** |  |

|  | **Species** | **Response variable** | **Predictor** | ***Estimate*** | ***SE*** | ***χ^2^*** | ***p*** | ***R^2^*** |
| --- | --- | --- | --- | --- | --- | --- | --- | --- |
| (a) | *Juncus bufonius* | Germination time (days) | Intercept | 2.596 | 0.051 |  |  | 0.027 |
|  |  |  | Faeces (addition) | -0.198 | 0.079 | 1.149 | 0.284 |  |
|  |  |  | Gut passage (ingested) | -0.151 | 0.056 | 2.362 | 0.124 |  |
|  |  |  | Gut x Faeces (ingested*addition) | 0.198 | 0.087 | 5.254 | **0.023** |  |
| (b) | *Eleocharis palustris* | Germination time (days) | Intercept | 2.632 | 0.053 |  |  | 0.168 |
|  |  |  | Gut passage (ingested) | 0.043 | 0.055 | 0.598 | 0.440 |  |
|  |  |  | Faeces (addition) | 0.305 | 0.046 | 44.599 | **<0.001** |  |

**Table S5**. Results from (a) *J. bufonius* (N = 323 seeds) and (b) *E. palustris* (N = 224) generalized linear models testing the effects of gut passage and faecal treatments on germination time*.* The *J. bufonius* model was fitted with a Poisson error distribution and the *E. palustris* model with a negative binomial error distribution, after detecting overdispersion in the Poisson model. Final models were selected based on likelihood ratio tests comparing models with and without a gut x faeces interaction (Table S2). Significant p-values are highlighted in bold. Predictor levels without ingestion or faeces addition are aliased in the model (i.e. estimates are effectively zero).

|  | **Species** | **Responsevariable** | **Predictor** | ***Estimate*** | ***SE*** | **F value** | ***p*** | ***R^2^*** |
| --- | --- | --- | --- | --- | --- | --- | --- | --- |
| (a) | *Juncus bufonius* | Above-ground mass (g) | Intercept | 0.347 | 0.021 |  |  | 0.218 |
|  |  |  | Germination time (days) | -0.007 | 0.001 | 24.910 | **<0.001** |  |
|  |  |  | Faeces (addition) | -0.073 | 0.009 | 65.176 | **<0.001** |  |
|  |  |  | Gut passage (ingested) | -0.021 | 0.012 | 3.134 | 0.077 |  |
| (b) | *Eleocharis palustris* | Above-ground mass (g) | Intercept | 0.185 | 0.042 |  |  | 0.301 |
|  |  |  | Germination time (days) | 0.022 | 0.005 | 18.134 | **<0.001** |  |
|  |  |  | Germination time (days)^2^ | -0.001 | <0.001 | 29.429 | **<0.001** |  |
|  |  |  | Faeces (addition) | -0.04 | 0.012 | 10.915 | **0.001** |  |
|  |  |  | Gut passage (ingested) | -0.006 | 0.013 | 0.221 | 0.638 |  |

**Table S6.** Results from (a) *J. bufonius* (N = 302 plants) and (b) *E. palustris* (N = 222) linear regressions examining the effects of time to germination, faeces and gut passage on above-ground plant biomass at collection time (final models selected based on the likelihood ratio test comparing models with and without gut x faeces interaction; Table S2). Shown are the parameter estimates and their standard errors for each continuous predictor and factor level. Significant p values are marked in bold. Predictor levels without ingestion or faeces addition are aliased in the model (i.e., estimates are effectively zero). Significant p-values are marked in bold.

| **Response variable** | **Predictor** | ***Estimate*** | ***SE*** | ***X^2^*** | ***p*** | ***R^2^*** |
| --- | --- | --- | --- | --- | --- | --- |
| Seeds per plant | Intercept | 9.012 | 0.164 |  |  | 0.067 |
|  | Faeces (addition) | -0.185 | 0.073 | 6.357 | **0.012** |  |
|  | Gut passage (ingested) | -0.130 | 0.105 | 1.586 | 0.208 |  |
|  | Germination time (days) | -0.029 | 0.010 | 7.791 | **0.005** |  |

**Table S7.** Results from the negative binomial model testing the effect of gut passage, faeces addition and time to germination on the total number of seeds per plant for *J. bufonius* (N = 189 plants). This final model was selected based on the likelihood ratio test comparing models with and without a gut x faeces interaction (Table S2). Significant p-values are marked in bold. Predictor levels without ingestion or faeces addition are aliased in the model (i.e., estimates are effectively zero).
